# Quantitative analysis of 3D extracellular matrix remodelling by pancreatic stellate cells

**DOI:** 10.1242/bio.017632

**Published:** 2016-05-11

**Authors:** Benjamin K. Robinson, Ernesto Cortes, Alistair J. Rice, Muge Sarper, Armando del Río Hernández

**Affiliations:** Cellular and Molecular Biomechanics Laboratory, Department of Bioengineering, Faculty of Engineering, Imperial College London, South Kensington Campus, London SW7 2AZ, UK

**Keywords:** 3D Biology, ECM remodelling, SHG, AFM

## Abstract

Extracellular matrix (ECM) remodelling is integral to numerous physiological and pathological processes in biology, such as embryogenesis, wound healing, fibrosis and cancer. Until recently, most cellular studies have been conducted on 2D environments where mechanical cues significantly differ from physiologically relevant 3D environments, impacting cellular behaviour and masking the interpretation of cellular function in health and disease. We present an integrated methodology where cell-ECM interactions can be investigated in 3D environments via ECM remodelling. Monitoring and quantification of collagen-I structure in remodelled matrices, through designated algorithms, show that 3D matrices can be used to correlate remodelling with increased ECM stiffness observed in fibrosis. Pancreatic stellate cells (PSCs) are the key effectors of the stromal fibrosis associated to pancreatic cancer. We use PSCs to implement our methodology and demonstrate that PSC matrix remodelling capabilities depend on their contractile machinery and β1 integrin-mediated cell-ECM attachment.

## Abstract (precis)

**Summary:** A 3D platform to characterise biomechanical remodelling of ECM by myofibroblast-like cells, using SHG microscopy, atomic force microscopy and quantitative algorithms to reveal structural, topological and mechanical properties of 3D matrices.

## INTRODUCTION

3D remodelling of the extracellular matrix (ECM), which involves changes in ECM rigidity and organisation, is integral to several biological processes, such as wound healing (Darby et al., 2014; Reinke and Sorg, 2012), fibrosis (Duscher et al., 2014; Ho et al., 2014), and embryogenesis, where mechanical forces dictate tissue organisation (Krieg et al., 2008). Additionally, in cancer, ECM rigidity promotes breast cancer progression via oncogenic signalling in epithelial cells (Levental et al., 2009), and tumour-associated fibroblasts remodel the ECM via Rho-dependent cytoskeleton contraction to facilitate cancer cell invasion (Calvo et al., 2013; Gaggioli et al., 2007; Goetz et al., 2011). In pancreatic ductal adenocarcinoma (PDAC), the strong fibrosis in the stromal region around the tumour is mediated via ECM remodelling and orchestrated by pancreatic stellate cells (PSCs) (Apte et al., 2011; Apte and Wilson, 2012; Olsen et al., 2011). Collagen alignment in the tumour periphery is used as a prognostic marker for survival in several cancers including breast cancer (Conklin et al., 2011), and it is known that highly aligned fibroblast derived matrices promote cancer cell invasion (Goetz et al., 2011). Assessing these quantitative changes in the ECM will provide a better understanding of the remodelling processes.

Due to the dearth of high-resolution microscopy, biophysical techniques and computer algorithms, until very recently our understanding of cells within their 3D environment was limited and based mostly on studies conducted with cells seeded on glass or 2D matrices. In these conditions, the mechanical and spatial cues from the environment sharply differed from the primary tissues of the cells under study (Cukierman et al., 2001). Significantly, some cells such as PSCs are culture activated on glass and most *in vitro* studies carried out with these cells fail to recapitulate the 3D environment or the tissue where these cells are quiescent. However, recent advances in image capture and analysis have opened a plethora of opportunities to study these cells in a 3D, physiologically relevant context.

In this work, we used atomic force microscopy (AFM) indentation, high resolution optical imaging, and custom made algorithms to provide a platform for the study of 3D matrix remodelling by cells. AFM indentation can be used to obtain the Young's modulus of samples through force spectroscopy. A glass micro-sphere attached to a cantilever indents the sample and is deflected in a manner determined by the sample stiffness, measured through deflection of a laser (Alexander et al., 1989). This method of obtaining a Young's modulus allows localised mechanical differences due to ECM remodelling to be detected that would not be obtained through larger-scale rheology studies. This remodelling can also be quantified by specifically imaging collagen-I, an abundant fibrous ECM component, using second harmonic generation (SHG) alongside multiphoton microscopy (MPM) (Acerbi et al., 2015; Campagnola and Loew, 2003; Raub et al., 2010). An optical signal for collagen-I can be obtained through SHG imaging with high specificity and without the need for immunostaining. The collagen topography can be analysed through existing as well as newly developed methods to correlate specific changes in ECM composition with changes in mechanical properties such as stiffness.

We used pancreatic stellate cells (PSCs) as a cell model for our analysis because they orchestrate PDAC associated fibrosis via ECM remodelling (Erkan et al., 2012), and these cells are highly sensitive to the effect of all-trans retinoic acid (ATRA), which render them to the quiescent-like state in which PSC remodelling ability is suppressed.

## RESULTS

### Quantification of matrix remodelling using SHG and immunofluorescence

**Fig. 1.**
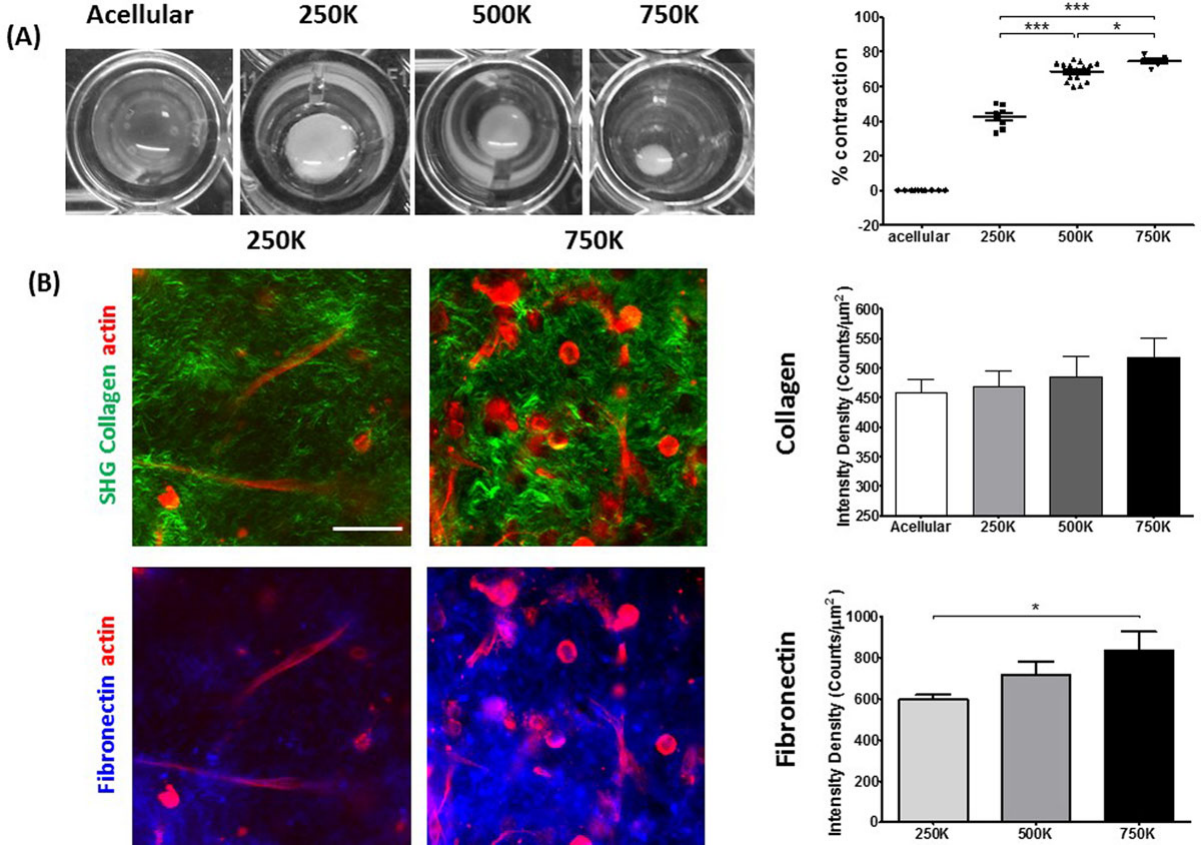
**Remodelling of 3D collagen-I matrices by PSCs.** (A) Bright-field images of matrix remodelling, assessed by matrix contraction (left). Mean±s.e.m. percentage change due to matrix contraction per cell number (right). Acellular, *n*=10; 250K, *n*=8; 500K, *n*=16; 750K, *n*=6. *P<0.05, ***P<0.0001 (unpaired *t*-test). (B) Immunofluorescence of PSCs (red), fibronectin (blue) and SHG imaging of collagen-I (green) for remodelled matrices containing 250,000 and 750,000 cells (left). Scale bar: 50 μm. Mean±s.e.m. of collagen-I and fibronectin intensity density of immunofluorescence (250K, *n=*13; 500K, *n*=11; 750K, *n*=7) and second harmonic signal for remodelled matrices (acellular, *n=*10; 250K, *n*=25; 500K, *n*=10; 750K, *n=*29) (right). Student's *t-*test shows a significant difference in fibronectin intensity between 250,000 and 750,000 cell conditions (*P=*0.01).

To assess the ECM remodelling capacity of PSCs, we prepared collagen/matrigel 3D matrices containing PSCs with increasing cell concentration. Monitoring the matrix contraction by imaging matrices in 96 well plates at 24 h intervals for 72 h allowed the assessment of the relative dimensional changes under remodelling. Taking the initial and final time points of the contraction, the percentage change in the matrix area represented the contractile ability of each condition. Matrix contraction was proportional to the number of seeded cells, with the maximum contraction of 80% observed for the matrices embedded with 750,000 cells (Fig. 1A;Fig. S1A).

**Fig. 2.**
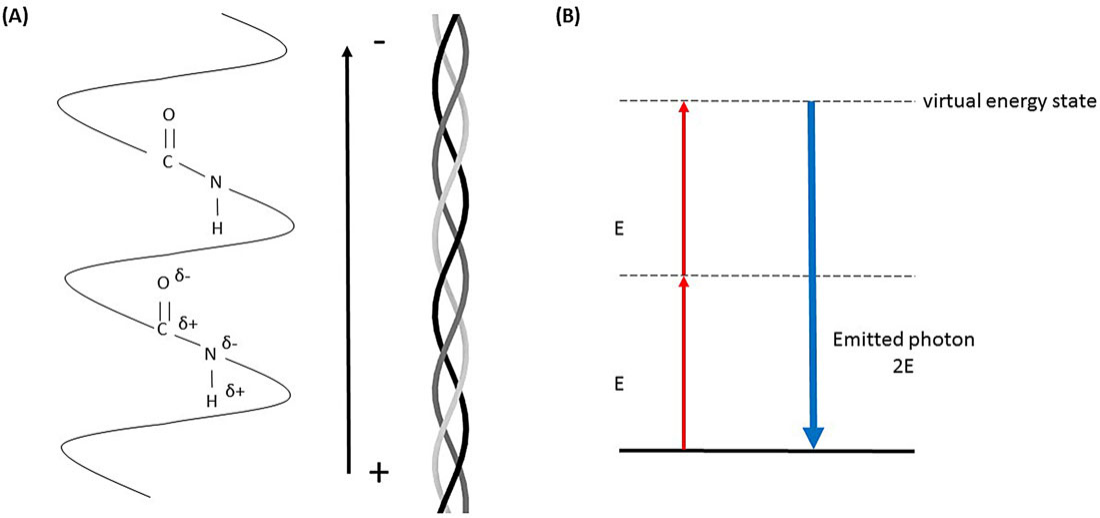
**Representation of SHG signal generation from collagen-I.** (A) The peptide bonds of the collagen chains create a permanent dipole moment along the triple helix that allows second harmonic generation. (B) Jablonski diagram of SHG. Excitation with two photons with identical energy E, leads to a virtual energy state and emission of a photon with energy 2E.

SHG is a microscopy technique that is highly responsive to fibrillar collagen (Chen et al., 2012). Collagen-I is a triple helix, made up of three α-helical chains, with these individual helices self-assembling into fibrils and larger-scale fibres. The peptide bonds linking together amino acids in the chains have their own dipole which, when amplified along the helix length of collagen-I, gives the fibrillar structure a permanent dipole moment (Fig. 2A). The lack of centrosymmetry that necessarily accompanies this gives collagen-l the optical properties required for SHG. The coherent process of SHG absorbs two identical low energy photons and emits one high energy photon of double the energy of the incident photons (Fig. 2B). This can only occur in a non-centrosymmetric molecule such as collagen-I (Campagnola, 2011). SHG benefits from only using endogenous species to provide contrast in measurement, preventing artefacts from use of exogenous agents (Chen et al., 2012), as well as decreased photobleaching and phototoxicity (Campagnola, 2011). Additionally, SHG can be performed on tissue sections hundreds of microns thick, which prevents artefacts and errors created from the cutting process, required for standard histology procedures (Campagnola, 2011).

SHG was used to visualise the effect of matrix remodelling on the collagen-I (hereafter collagen) structure and topology. Intensity density of the collagen in regions of interest shows a slight, albeit non-significant, increase to the collagen signal with increased cell number (Fig. 1B), which may arise from the increased cell number and the greater impact that this has on matrix remodelling. SHG microscopy imaging of collagen in the matrices allowed for assessment of the percentage of collagen present in a region of interest. The ratio between the number of collagen containing pixels and the total pixels in a region of interest show that remodelled matrices, when compared to acellular conditions, contain more collagen. This increase can indicate that more collagen is drawn into the imaging plane when matrix remodelling is present (Fig. S2B).

Immunostaining was carried out to visualise fibronectin, another major ECM protein secreted by PSCs, which is known to promote fibrosis, favouring PDAC dissemination to distant sites (Costa-Silva et al., 2015). Across the regions of interest, this shows an increase in the intensity density of fibronectin with increased cell number. Through visual observation of the fibronectin staining, the intensity is high in the vicinity of cells, corresponding with the regions of higher collagen remodelling (Fig. 1B). Colocalisation analysis was conducted on the fibronectin/collagen channels to ensure that light leakage between channels was not responsible for the signals. This analysis yields a Pearson coefficient of ∼0.3, suggesting that the signal in the fibronectin channel is not due to collagen and vice versa (data not shown).

### Remodelling induces topological and structural changes to collagen matrices

**Fig. 3.**
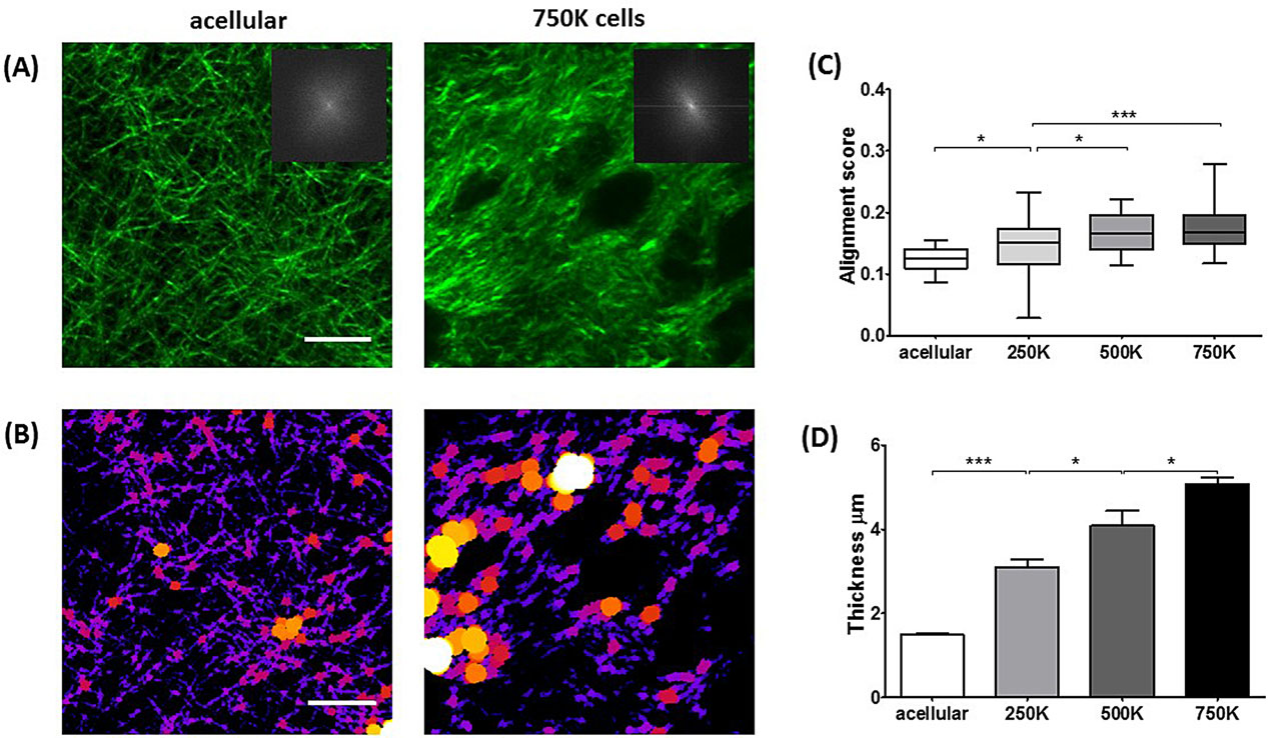
**ECM remodelling by PSCs modifies collagen topology/structure.** (A) SHG images of collagen-I in matrices. Insets show FFTs of collagen-I images, representing alignment with respect to the elliptical distribution of the FFT central maxima. (B) Images in A represented through the BoneJ plugin to calculate fibre thickness where larger spheres fit along fibres represent greater thickness. (C) In the box-and-whisker plot, the central box represents values from the lower to upper quartile. The middle line represents the mean. The vertical line extends from the minimum to the maximum value. Values represent degree of collagen-I alignment where higher score between 0-1 represents more aligned structures. Acellular, *n=*14; 250K, *n=*30; 500K, *n=*16; 750K, *n=*30. *P<0.05, ***P<0.0001 (unpaired *t*-test). (D) Collagen-I thickness. Acellular, *n=*10; 250K, *n=*23; 500K, *n=*10; 750K, *n=*27. Values are represented as mean±s.e.m. *P<0.05, ***P<0.0001 (unpaired *t*-test). Scale bars: 20 μm.

Collagen topography can affect the survival and progression of cancer cells, and quantification of structural changes is therefore required. (Conklin et al., 2011; Goetz et al., 2011). Using fast Fourier transforms (FFTs) to acquire a representation of the angular frequencies within a region of interest, we have been able to develop an objective analysis to quantify collagen fibre alignment in the presence of ECM remodelling. Alignment in the collagen network manifests as a more elliptical distribution of the central maxima in the FFT power spectrum orthogonal to the direction of collagen alignment. Greater elliptical eccentricity of this power spectrum relates to a higher degree of alignment (Zhuo et al., 2010) (Fig. 3A, insets). In Matlab, we have developed a program to acquire FFTs and produce radial intensity histograms to indicate alignment (Jones et al., 2005). For each 1° angle over 180°, rotating about the centre of the image, the sum of pixel intensities is calculated and plotted against the angle (Fig. S3). The spread of the distribution of pixel intensities acquired relates to the alignment, where a tighter distribution profile indicates a more elliptic FFT. The reciprocal of the distribution (standard deviation) is used as an alignment score where a higher number relates to more alignment (Fig. 3C). We observed significant increases in collagen fibre alignment as the PSC number in the matrix increased.

To further investigate the effects of ECM remodelling by PSCs on the collagen topography we have quantified the width of collagen fibres using the BoneJ plugin for ImageJ (Doube et al., 2010). To calculate the thickness of fibres the plugin fits a sphere along the fibres in an image that has had a threshold applied, and takes the thickness as the average largest diameter circle that will fit within the fibre. Measurement of the collagen fibre thickness shows significant increases that scale with the increase in matrix contraction (Fig. 3D). The increase of collagen fibre thickness with increased matrix remodelling capacity may be a result of the remodelling on the thinner collagen fibrils (individual fibrils ∼200 nm; Kadler et al., 1996). Remodelling may lead to more fibrils being drawn into collagen fibres (bundles of fibrils) leading to an increase in fibre thickness due to the packing of a greater number of fibrils. Bundling of more collagen into fibres may also account for the observed increasing in spacing between fibres (also calculated through BoneJ) (Fig. S2A).

### Quantification of ECM stiffness using AFM indentation

**Fig. 4.**
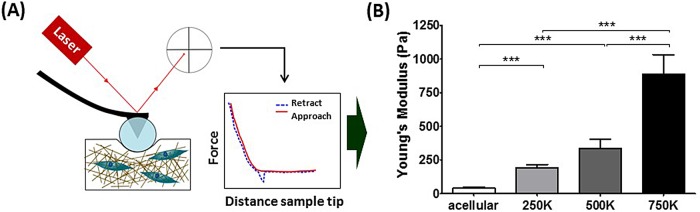
**ECM remodelling by PSCs induces matrix stiffening.** (A) Schematic of AFM indentation. Cantilever with a glass bead indents the remodelled matrices and detection of the reflection of a laser on the cantilever head by a photodiode allows the acquisition of force-distance curves from where the Young’s modulus was calculated and plotted in B. (B) Young's modulus values for the remodelled matrices, values are mean±s.e.m. Acellular, *n=*58; 250K, *n=*51; 500K, *n*=97; 750K, *n*=44. ***P<0.0001 (unpaired *t*-test).

To characterise the stiffness of the remodelled matrices we used AFM to acquire the Young's modulus through force spectroscopy. AFM is used to determine substrate stiffness in a localised manner by indentation. A cantilever with a 70 μm glass bead attached to the tip is positioned a few microns above the sample, and reflects a laser onto a photodiode. This setup approaches the surface and makes contact, indenting the matrix (Fig. 4A). The cantilever acts as a spring and is deflected by sample contact in a manner dependent on the stiffness. The laser reflected off the cantilever is also deflected and moves its position on the photodiode, which records the alterations as a voltage. Using calibrated values for tip sensitivity and cantilever spring constant, this voltage is converted to the force applied, and plotted against the displacement of the setup during indentation to produce a force-displacement curve (Thomas et al., 2013). The Hertz contact model is applied to the approach phase of the force curve to produce a measure for the Young's modulus, which is indicative of stiffness (Harris and Charras, 2011). AFM has high sensitivity, and due to its localised nature of indentation, high spatial resolution which allows correlation of stiffness measurements with collagen structure obtained through SHG microscopy that cannot be achieved with larger scale rheology measurements (Kirmizis et al., 2010).

The Young's moduli of these matrices show significant increases correlating to the increasing number of cells present in the matrices, when compared to the acellular matrices (Fig. 4B). This is in agreement with the previous data that showed increased alignment and thickening of the collagen fibres alongside changes in matrix remodelling with increasing cell number. This data shows that the stiffening applies throughout the matrix and not just on the planes observed in the analysis of the collagen SHG signal.

### The capability of PSCs to remodel the ECM depends on the contractile apparatus

**Fig. 5.**
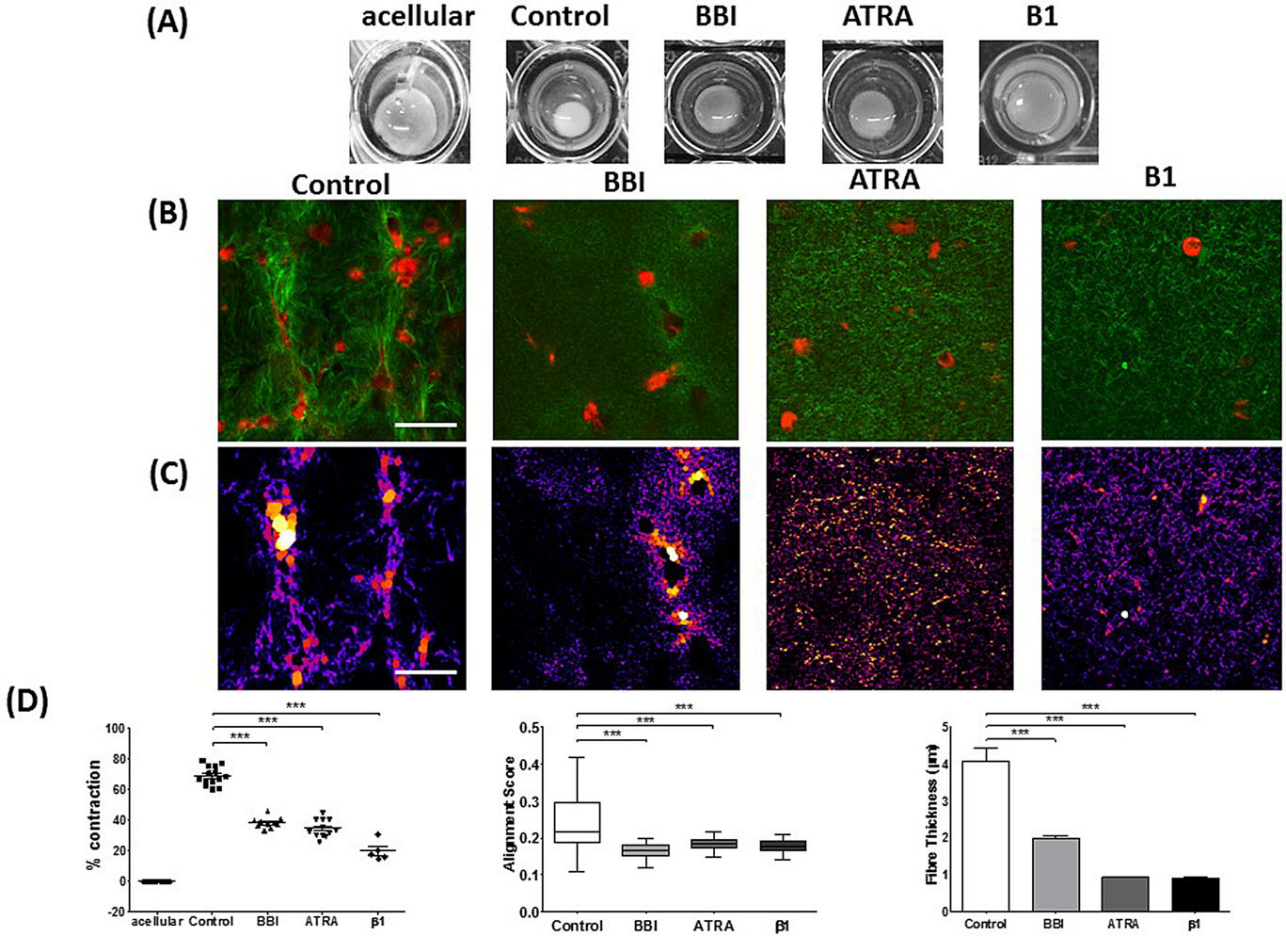
**PSCs’ ability to remodel ECM is dependent on actomyosin machinery and activation state.** (A) Bright-field images of matrix remodelled by PSCs (control, BBI, ATRA and β1 integrin inhibition). (B) Immunofluorescence of PSCs (red) and SHG imaging of collagen-I (green) for remodelled matrices. (C) Images in B represented through the BoneJ plugin to calculate fibre thickness where larger spheres fit along fibres represent greater thickness. (D) Graphs of matrix contraction, collagen alignment and collagen thickness, respectively. Each point represents a matrix for percentage contraction. Acellular, *n*=10; control, *n=*14; BBI, *n*=10; ATRA, *n=*12; β1, *n*=5. In the box-and-whisker plot for alignment, the central box represents values from the lower to upper quartile, the middle line represents the mean. The vertical line extends from the minimum to the maximum value. For alignment: control, *n=*28; BBI, *n*=20; ATRA, *n*=78; β1, *n*=40. Histograms of fibre thickness are plotted as mean±s.e.m. Control, *n=*10; BBI, *n=*50; ATRA, *n=*20; β1, *n*=30. ***P<0.0001 (unpaired *t*-test). Scale bars: 50 μm.

The ability of myofibroblasts to remodel the matrix relies on the ability to apply contractile forces (Calvo et al., 2013; Goetz et al., 2011). To gain more insight into the mechanisms underlying the ECM remodelling capacity by PSCs, we analysed collagen topology and structure in the presence of blebbistatin (BBI) and all-trans retinoic acid (ATRA). BBI blocks myosin II ATPase activity, and thus actomyosin contraction (Kovacs et al., 2004), and ATRA is the active metabolite of vitamin A and induces quiescence on PSCs (Froeling et al., 2011). Inhibition of actomyosin contraction in PSCs using BBI or inducing quiescence with ATRA significantly reduced the capacity of PSCs to contract the matrix (Fig. 5A,D). In both cases, the collagen alignment score and the fibre thickness significantly reduced with respect to the control condition (Fig. 5A-D; Fig. S1B). A significant decrease in the collagen intensity was also noted within the matrix embedded with PSCs treated with BBI and ATRA (Fig. S2C). We further observed a significant decrease in the spacing between collagen fibres in the matrices treated with BBI and ATRA (Fig. S2E) Taken together these results indicate that PSCs' capacity to remodel the matrix is only present in the active state and depends on their actomyosin machinery.

To investigate the ability of the PSCs to adhere to the ECM and their ability to sense and apply force to the ECM, we blocked β1 integrin activity. β1 integrin is required to allow cells to form focal adhesions and to form bonds with the ECM, which are required for force application and sensing (Roca-Cusachs et al., 2009). Under the inhibition of β1 (through a β1 integrin blocking antibody) it is observed that ECM remodelling is significantly reduced as confirmed by the reduced matrix contraction (Fig. 5A,D). In accordance with previous trends observed, when β1 integrin was inhibited, we saw a reduction in the collagen alignment score and in the fibre thickness, compared to the control condition. These results indicate that by arresting the ability of cells to adhere to the ECM and thus apply force, remodelling is inhibited and the presence of cells in matrices does not affect the collagen topology (the effects on collagen come from the contractile mechanisms).

## DISCUSSION

Here, we report a method that blends high resolution microscopy with biophysical and modelling approaches for the accurate characterisation of the capacity of myofibroblast-like cells to remodel 3D matrices. SHG microscopy was used to visualise the collagen structure and topology. Our approach to visualising collagen with the SHG signal that comes from its non-centrosymmetric properties allows for a highly specific emission signal to be observed. Minimal background signal is observed due to no requirement for staining, giving no risk of non-specific staining of collagen or staining effects on collagen architecture. To quantify the changes to collagen observed in the SHG images we have employed image analysis techniques to measure alignment and thickness of collagen fibres. These techniques are not dependent on the intensity of the collagen signal, and therefore avoid any subjectivity in the measurement, as SHG intensity could be dependent on collagen substructure, such as cross-linking (Lutz et al., 2012).

Our approach to the assessment of collagen structure has led to the development of an algorithm to quantify the alignment score for the collagen fibres present in selected regions of interest in the SHG images acquired for the matrices. Quantification of collagen fibre alignment through automated methods lacks the subjectivity of visual analysis, as it removes bias and improves reproducibility (Frisch et al., 2012). Many methods such as edge detection (Kemeny and Clyne, 2011), fractal analysis (Frisch et al., 2012), and two-dimensional fast Fourier transform (2D-FFT) exist for alignment quantification. Previous techniques to analyse fibre orientation fit an ellipse to the central region of a 2D-FFT and quantify the alignment as related to the eccentricity of this ellipse (Frisch et al., 2012; Sereysky et al., 2010; Zhuo et al., 2010). The ellipse fitting method can be sensitive to noise within the image and can suffer from a non-uniqueness of solutions (Fitzgibbon et al., 1999); furthermore, noise elements can affect the determination of the ellipse short axis which can severely affect the eccentricity calculation especially where this axis is very small (e.g. where the ellipse has very high eccentricity). Alternatively, we have used a Matlab code to produce radial intensity histograms from the FFT power spectrum to produce an alignment score, which is defined as a product of the inverse of the standard deviation of a Gaussian function fit to this curve. The analysis we have employed removes the effect that noise has on the FFT and on the alignment calculation in the Gaussian curve fitting stage as more of the FFT image has been taken into account in the radial summation so noise constitutes a smaller percentage of the fit value.

We have furthered the analysis of alignment of collagen to incorporate the effect that this has on the stiffness of the ECM through the use of AFM indentation. These investigations have shown that significant increases in matrix stiffness occur in the conditions that present as being more greatly remodelled and showing a greater degree of collagen alignment and fibre thickness. These results follow from some observations that have seen greater alignment in regions around tumours which manifest as also being stiffer.

We have used these techniques and algorithms to characterise for the first time the behaviour of PSCs in 3D matrices that mimic the physiological conditions of diseased pancreatic tissues. Our data show that PSCs are able to remodel the ECM via matrix contraction and increasing collagen fibre alignment and thickness. We have also observed that the PSCs’ ability to remodel the ECM rely on their contractile actomyosin machinery and on integrin mediated cell-ECM attachment. Rendering PSCs to their quiescent state abolishes their capacity to remodel the ECM.

The experimental techniques that we have applied and the analysis techniques we have developed in this work can be applied to other matrices or cell systems, as well as to tissue samples. For instance, these techniques can be used with collagen matrices in organotypic experiments to examine the cell-cell and cell-ECM interactions in these 3D matrices. It may also be possible to use SHG imaging of collagen to quantify the changes to matrices after invasion; this may also be coupled with immunofluorescence for the analysis of cells. These techniques can also be applied to tissue samples to quantify stiffness with AFM indentation and the arrangement of collagen using the analysis of SHG images. In this case tissue cubes can be used for AFM and tissue cryosections can be used for SHG analysis with comparisons between different stages of fibrosis.

## MATERIALS AND METHODS

### Cell culture

Primary, culture-activated human pancreatic stellate cells (passage 5-8 PSCs, HPaSteC, #3830, ScienCell, CA, USA) were cultured for at least two media changes and until cells reached a confluency between 65% and 75%. Cells were incubated with Dulbecco's Modified Eagle's Medium (DMEM-F12HAM) (Sigma-Aldrich, UK), with 10% Foetal Bovine Serum Heat Inactivated (FBS) (Gibco, UK), 1% penicillin/streptomycin (Sigma-Aldrich, UK), and 1% fungizone Anphotericin B (Gibco, UK). For the all-trans retinoic acid (ATRA) (#R2625, Sigma-Aldrich, UK) treatment condition, cells were exposed to ATRA dissolved in ethanol at a concentration of 1 μM for 10 days with medium changed every 24 h and treatment performed in subdued light. A control PSC group for ATRA treatment was established by adding 1 μl/ml of ethanol to the control media during the 10 days.

### 3D ECM remodelling assay

To analyse the ECM remodelling ability of PSCs, Collagen type-I, High Concentration, Rat Tail (Collagen-I; Corning, #354249, stock concentration 8.96 mg/ml) and Matrigel^®^ Matrix Basement Membrane (Corning #354248, stock concentration 9 mg/ml) mixture gels were prepared with 1:10 10× DMEM (Sigma-Aldrich, UK) and 1:10 FBS, yielding to a final concentration of 4.5 mg/ml Collagen-I and 2 mg/ml Matrigel. The gel mixture was neutralised with 1 M NaOH (Sigma-Aldrich, UK) and the desired number of cells in culture media were added to the gel mixture. 80 µl gel volume were added to wells in a 96-well plate, which was pre-treated with 3% BSA (Sigma-Aldrich, UK) for 1 h, washed with Dulbecco's phosphate buffered saline (PBS) (Sigma-Aldrich, UK) and air dried for 10 min. Gels were set at 37°C for 1 h and incubated with culture media for 72 h at 37°C (media change conducted every 24 h). For the InSolution™ Blebbistatin Racemic (BBI) (#203389, CalBiochem, CA, USA) treatment condition, gels with the desired number of cells were exposed to BBI at a concentration of 20 μM with media changed every 24 h. For the ATRA treatment, gels with the desired number of cells were exposed to ATRA at a concentration of 1 μM with media changed every 24 h. The media of the established control PSC group for ATRA was replaced every 24 h with fresh control media supplemented with 1 μl/ml of ethanol. Both BBI and ATRA media changes were performed in subdued light. To investigate the role of β1-integrin activity in the PSCs ability to remodel the matrix, β1-integrin mediated cell-ECM attachment was blocked by adding 1 µg/ml β1-integrin function blocking antibody (clone:BV7, ab7168, Abcam, UK). The β1-integrin was added in the media 30 min before the desired amount of PSCs were embedded in the matrices. Media was changed every 24 h and the blocking antibody was present in the media for the 72 h incubation period where cells could remodel the matrices. For SHG (gels for AFM remained unfixed), remodelled gels were fixed with 4% paraformaldehyde (PFA) (Sigma-Aldrich, UK) in PBS for 1 h at 37°C, then washed with PBS and permeabilised with 0.3%Triton X-100 (Sigma-Aldrich, UK) in PBS for 30 min. Gels were then blocked with 1% BSA 0.1% Triton X-100 in PBS for 1 h. After blocking, cells were incubated with a primary antibody (anti-fibronectin antibody, #ab2413, Abcam, UK) prepared in blocking solution for 1 h. Gels were washed with PBS and stained with goat anti-rabbit Alexa Fluor^®^488 (#A11030, Life Technologies, CA, USA) conjugated secondary antibody- and Phalloidin- conjugated to orange-fluorescent Alexa Fluor 546 dye (#A22283, Invitrogen, CA, USA) at 1/300 dilution in 1% BSA in PBS for 30 min. Finally gels were washed two times with PBS. At least *n*=5 gels were used for each condition across at least two gel preparations (repeats).

### Atomic force microscopy

Collagen matrices were lifted from the 96-well plates prior to measurement and immediately attached to a petri dish with a droplet of cyanoacrylate adhesive, applied with a 10 μl pipette tip. After matrix attachment (1-2 min) the matrix was immersed in culture medium (DMEM with 2% FBS) for AFM measurements to be conducted within a 2 h time period. Matrix measurements were conducted on a JPK Nanowizard-1 (JPK Instruments, Germany) operating in force spectroscopy mode, mounted on an inverted optical microscope (IX-81; Olympus, Japan). AFM pyramidal cantilevers (MLCT; Bruker, MA, USA) with a spring constant of 0.07 N/m were used with a 70 μm diameter glass bead attached to cantilever tip. Prior to measurements with the adapted cantilevers, their sensitivity was calculated by measuring the slope of force-distance curve in the AFM software on an empty region of the petri dish. For indentation tests, the cantilever was aligned over regions in the middle of the sample of interest using the optical microscope and for each matrix 30 force curves were acquired across 6 different 100 μm regions. This arrangement allowed force-curves to be acquired in locations at least 50-100 μm apart. Force-curve acquisition was carried out with an approach speed of 5 μm/s and a maximum set force of 1 nN. Elastic moduli were calculated from the force-distance curves by fitting the contact region of the approach curve with the Hertz contact model (Harris and Charras, 2011), using the AFM software (JPK).

### Multiphoton confocal microscopy

Collagen matrices were prepared for analysis on petri dishes via the same method mentioned previously for AFM analysis. All SHG images were obtained using a custom built multiphoton microscope, incorporating an upright confocal microscope (SP5, Leica, Germany) and a mode-locked Ti:Sapphire Laser (Mai Tai, Newport Spectra-Physics, UK). Images of the SHG signal from collagen-I were collected using an 860 nm excitation with SHG signal obtained with a 414/46 nm bandpass filter and multiphoton autofluorescence signal obtained with a 525/40 nm bandpass filter. A 25×, 0.95 NA water-immersion objective (Leica) was used to deliver excitation signal and to collect the SHG emission signal from the sample. Images with a 620 μm×620 μm field of view were obtained with 2048 pixel resolution and a line rate of 10 Hz giving a pixel resolution of ∼0.3 μm with 3× averaging on each acquisition to reduce the effect of noise. Further to this a 488 nm Argon+ and a 543 nm HeNe laser were used for excitation of Alexa Fluor 488 and Phalloidin with emission filters at 520 nm and 580 nm used for collection, respectively.

### Analysis of multiphoton images

SHG images obtained through multiphoton microscopy were analysed to quantify collagen properties of matrices after remodelling. For the assessment of collagen concentration, At least four SHG images obtained for collagen in each condition were analysed in ImageJ (NIH) to acquire intensity density across fields of view of 200 μm, taking five regions of interest from each full field of view (620 μm). Intensity density values from were analysed with the software Prism (GraphPad) for histogram representation. Student's *t*-tests were conducted on the datasets showing the significant differences between the datasets.

For the analysis of collagen fibre organisation, 200 μm field of view images were used for intensity density analysis, with each image split into 4×100 μm images in ImageJ. Using a custom MatLab program, images were converted to a 2D Fourier transform power plot. In the program a circular projection is positioned in the centre of this symmetrical image and the image converted to an array containing the sum of pixel intensities along the radii of the circle from 0°-180°. A Gaussian is fit to the array where an alignment score is calculated as the inverse of the standard deviation of the curve where values between 0-1 relate to alignment, where increasing value indicate greater degree of alignment. Significance was measured through Student's *t*-tests in Prism (GraphPad).

Collagen presence was observed in ImageJ by applying thresholding to highlight the collagen containing pixels and then the observation of the histogram of pixel values. The number of collagen pixels could be obtained and divided by the total number of pixels (in the 200 μm images) and then data could be displayed in GraphPad with analysis via *t*-tests.

Fibre thickness was calculated with the BoneJ plugin for ImageJ (http://bonej.org/). Thresholded images were run through the plugin which obtained an average fibre thickness value for the image (200 μm) where this average thickness value was exported to Prism for analysis and significance calculations via the Student's *t*-test.

Fibre spacing was calculated through the BoneJ plugin in ImageJ. Using the same images for the fibre thickness analysis where threshold had been applied, the average collagen spacing was calculated by the same process as thickness where a sphere is fit in the region without collagen and the diameter relates to the spacing. Significance was calculated via Student's *t*-test in Prism.
